# Clinical, Phenotypic, and Demographic Characteristics of Peruvian Children and Neonates with Autosomal and Sex Chromosome Aneuploidies

**DOI:** 10.5152/eurasianjmed.2023.22070

**Published:** 2023-02-01

**Authors:** Jeel Moya-Salazar, Víctor Rojas-Zumaran, Rafael Vega-Vera, Eduardo Espinoza-Lecca, Hans Contreras-Pulache

**Affiliations:** 1 School of Medicine, Universidad Norbert Wiener Faculties of Health Science, Lima, Peru; 2 Department of Pathology, Hospital Nacional Docente Madre Niño San Bartolomé, Lima, Peru; 3 Genetic Unit, Nesh Hubbs, Lima, Peru; 4 South America Center for Research and Public Health, Universidad Norbert Wiener, Lima, Peru

**Keywords:** Aneuploidy, Down syndrome, trisomy 21, Edwards syndrome, Turner’s syndrome

## Abstract

**Objective:**

Autosomal and sex chromosome aneuploidies are associated with multiple risk factors that determine their frequency and their social and health impact. We aimed to determine the clinical, phenotypic, and demographic characteristics of Peruvian children and neonates with autosomal and sex chromosome aneuploidies.

**Materials and Methods:**

This was a retrospective study conducted on 510 pediatric patients. We conducted a cytogenetic analysis with G-bands by trypsin using Giemsa (GTG) banding, and the results were reported using the International System for Cytogenetics Nomenclature 2013 system.

**Results:**

Of 399 children (mean age 2.1 ± 4 years), 84 (16.47%) had aneuploidies, with 86.90% being autosomal (73.81% trisomies). In autosomal aneuploidies, 67.85% (n = 57) of the children had Down syndrome where the most common cause was free trisomy 21 (52 cases, 61.91%), followed by Robertsonian translocation (4 cases, 4.76%). Edwards and Patau syndrome affected 4 (4.76%) and 1 (1.19%) neonate. The most frequent phenotypic characteristics in children with Down syndrome were Down syndrome-like facies (45.61%) and macroglossia (19.29%). Of sex chromosome aneuploidies, 6/7 were abnormalities of the X chromosome (mainly 45,X). Neonate’s age (19 ± 44.9 months), paternal age (49 ± 9 years), height (93.4 ± 176 cm), and gestational age (30 ± 15.4 weeks) were significantly correlated with the presence of sex chromosome and autosomal aneuploidies (*P* < .001; *P* = .025; and *P* = .001).

**Conclusions:**

Down syndrome and Turner’s syndrome were the most frequent aneuploidy and sex chromosome aneuploidy, respectively. In addition, some of the clinical, phenotypic, and demographic characteristics, such as newborn’s age, paternal age, gestational age, and height, were significantly correlated with the occurrence of aneuploidy. In this sense, these characteristics could be considered risk factors among this population.

## Introduction

**Main Points**

- We determined the frequency of 16.47% of children with aneuploidy, with autosomal alterations being the most common.
- Trisomies are the most common autosomal aneuploidy variant, with Down syndrome affecting 67% of cases.
- All sexual aneuploidies affected the X chromosome, where 45,X was the most frequent karyotype.

Aneuploidies correlated with the neonate’s age (19 ± 44.9 months) and paternal age (49 ± 9 years).

Trisomies represent one of the main causes of congenital diseases worldwide, with Down syndrome (DS) being the most common disease of this kind and the leading cause of intellectual disability in the United States (a prevalence of 1 per 792 live births).^1^ In the United Kingdom, it is estimated that 1 in 1000 live births is affected,^2^ while in Latin America no statistics are available, although their disability-adjusted life years are estimated at 59.1 per 100 000 inhabitants.^3^

The genetic cause of DS is trisomy 21 (3 copies of chromosome 21). People with this disorder develop a variety of medical conditions, including eye problems, mental retardation, congenital cardiovascular diseases, decreased thyroid function, and obesity.^4,5^ Despite these complications, it has been observed that a child with DS costs US$2.77 more per day than a child without this syndrome, which suggests that the families with a child with DS do not represent a financial burden excessive in high-income countries.^6^ However, this situation may be different in low- and middle-income countries, where health systems are unable to meet the necessities of patients with DS and their families.^7,8^

Since 95% of trisomy 21 cases are due to maternal chromosomal nondisjunction, maternal age (≥35 years) is the main factor associated with this anomaly.^9,10^ However, there are other factors, such as reproductive performance, father’s age, maternal periconceptional tobacco use, and long-standing use of oral contraceptives, which could be related to the development and prognosis of the DS.^11,12^

Other kinds of aneuploidy, such as Edwards syndrome (trisomy 18) and Patau syndrome (trisomy 13), are the most common, after DS. Trisomy 18 is the second most common autosomal aneuploidy after DS (a prevalence of 1 per 2500 live births). In this syndrome, the prevalence increases with maternal age.^13^ Conversely, trisomy 13 is the least common (incidence between 1.3 and 1.9 per 10 000 live births), but it is the most severe viable autosomal trisomy (average survival ≤3 days) since only 1 in 20 births survive more than 6 months.^14,15^

In the region of the Americas, there may be differences in the frequency of trisomies, affecting the neonatal and infant population at different levels. In this sense, understanding the frequency and the clinical and epidemiological characteristics associated with these aneuploidies is key to facilitating prevention and maintenance activities for patients with these changes and their relatives. We aimed to determine the clinical, phenotypic, and demographic characteristics of Peruvian children and neonates with autosomal and sex chromosome aneuploidies.

## Materials and Methods

### Study Design, Population, and Clinical Assessment

The study population consisted of all pediatric and adolescent patients (newborns up to 18 years, inpatient or outpatient) at Hospital Nacional Docente Madre Niño San Bartolomé (Lima) in the period from January 1 to December 31, 2015, and referred to the Pathological Anatomy Service of the Diagnostic Aid Department for cytogenetic analysis due to clinical suspicion of chromosomal alteration (N = 610). This hospital is a quaternary care public hospital which belongs to the Ministry of Health of Peru and specializes in attending mothers and children through several agreements with Health Networks. Patients who did not voluntarily agree to participate in the study and did not sign the informed consent, or whose parents/legal representatives did not consent to their participation and patients whose blood samples were <3 mL, were incomplete, or apparently contaminated (n = 100) were excluded from the study. After applying these exclusion criteria, 510 patients remained in the analysis.

The recruitment of the participants was carried out directly through an in-house call during the medical consultation, where information was provided to the patients to be included in the study. The minimally invasive diagnostic approach included an interview and analysis of chromosomal abnormalities. The clinical indication for the study included advanced maternal age, possible hereditary disease affecting the fetus, abnormal serum detection of suspect markers (i.e., alpha-fetoprotein), known or suspected fetal anomaly, and poor prior pregnancy history (i.e., previous aneuploid pregnancy).

### Clinical, Phenotypic, and Demographic Characteristics

An interview was conducted by a pathologist with each included patient (n = 510) or the patient’s legal representative. Also, clinical (e.g., personal medical history, family history, type of delivery, premature birth, paternal and maternal age, and number of siblings), phenotypic (e.g., height and weight), and demographic (e.g., age and sex) information was obtained (study variables) . The interview was conducted according to the hospital procedures, using a data collection instrument and a semistructured interview, as reported in other studies.^16^ Study risk variables included in the study were advanced maternal age (>30 years), previous miscarriage, abnormal fetal ultrasound, or screening for positive serum markers (i.e., human chorionic gonadotropin and alpha-fetoprotein)

### Cytogenetic Analysis

The chromosomal abnormality analysis was focused on the identification of autosomal and sex chromosome aneuploidies. According to the standardized operational procedure of the Cytogenetics and Molecular Biology Area of the hospital, a 4 ± 1 mL sample of venous blood was taken from each patient, using a syringe preloaded with 0.1 mL sodium heparin as anticoagulant to carry out the chromosome culture.^17,18^ The samples were processed immediately after collection or stored under refrigeration at 1°C for a maximum of 3 ± 1 hours prior to processing.

Peripheral blood lymphocyte cultures were grown in PB-MAX™ Karyotyping medium (Thermo Fisher Scientific, Waltham, Mass, USA) and in KREAvital Lymphocyte Karyotyping (Kreatech Bio., Amsterdam, Netherlands) and were incubated at 37 ± 1°C for 48 hours. The harvest method was conventional, starting with Colchicine (colcemid 0.1 ug/mL ), followed by hypotonization with potassium chloride at 0.075 M, until fixation with commercial Carnoy’s solution. To identify the karyotype, the metaphase plaques were manually analyzed under a light microscope using GTG banding with Giemsa staining (modified from Leishman’s); then, the fields with karyotypes were selected with the Leica X210 System analyzer (Leica, Wetzlar, Germany).^19^

The International System for Cytogenetics Nomenclature (ISCN) version 2013 was used to report the results of cytogenetic analyses within 15 days after performing the culture. During the karyotype reading, a new sample was taken from those patients with few metaphases (n = 22) (≤5 metaphases in all preparations) or without open metaphases (n = 11) to realize a new evaluation.

### Statistical Analysis

All data were tabulated in a matrix created in MS-Excel 2013 (Redmond, Wash, USA). A descriptive analysis of the data was realized, considering absolute frequencies and percentages for the categorical variables and means and standard deviations for the quantitative variables. With regard to the inferential analysis, the chi-square test was used to determine the differences between variables and the correlation between these variables. The presence of autosomal and sex chromosome aneuploidies and the type of aneuploidy were evaluated using the Spearman correlation coefficient. A significance level of *P* < .05 and a 95% CI were considered for all statistical analyses. The data were analyzed using the Statistical Package for Social Sciences software v25.0 (IBM SPSS Corp.; Armonk, NY, USA) for Windows.

### Ethical Approval

The present study followed the ethical principles for conducting biomedical research involving human beings from the Declaration of Helsinki.^20^ In addition, informed consents were obtained from the patients’ legal representatives. The study was approved by the Hospital Nacional Docente Madre Niño San Bartolomé Ethics Committee according to registry no. 16913-16-OADI-HONADOMANI-SB, dated December 22, 2016.

## Results

Participants presented a mean age of 2.1 ± 4 years (95% CI, 1.1-3.1). Two hundred fifty-seven of the included patients (50.39%) were girls. No significant difference was found between the mean age of girls and boys (2.3 ± 4.5 and 1.7 ± 1 years; *P* = .009). Three hundred ninety-nine (78.23%) had normal karyotypes and 84 (16.47%) had autosomal and sex chromosome aneuploidies. In 27 patients (5.29%), it was not possible to obtain culture results due to lack of growth or contamination of the culture medium (cases excluded).

**Figure 1. A-L.**
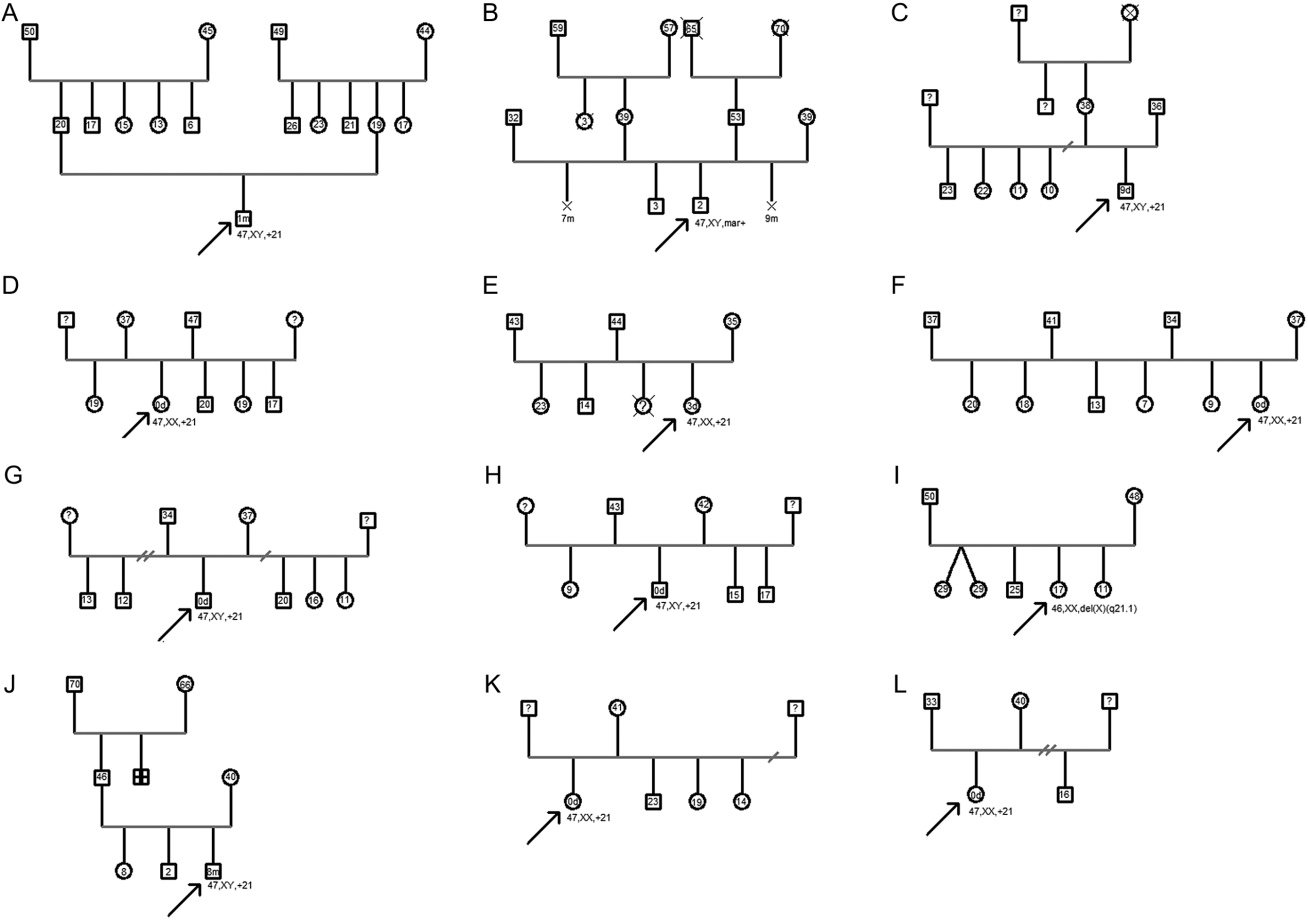
Main genealogies of 49 children with aneuploidies in Lima, Peru. We have described Down syndrome males (A, C, G, H, J) and females (D, E, F, K, L), with trisomy 13 and other autosomal (B) and sexual aberrations (I). In each case, family tree does not cover more than 2 degrees of consanguinity; evaluators did not include other relevant epidemiological data from the history of the patients. In a single case, detailed information is shown (A). The purpose of the study is indicated by black arrows. Software: GenoPro v3.0 (GenoPro Inc, York, Maine., USA).

Of the 84 children with aneuploidies, genealogical data were obtained in 49 (58.33%) (Figure 1). Of the 84 cases, 11 were sex chromosome aneuploidies (13.09%) and 73 autosomal (86.90%). With respect to autosomal aneuploidy, 67.85% (n = 57) of the children had DS where the most common cause was free trisomy 21 presenting in 52 cases (61.91%), followed by Robertsonian translocation in 4 cases (4.76%). Edwards and Patau syndrome affected 4 (4.76%) and 1 (1.19%) children, respectively. The total percentage of free trisomies that affected chromosomes 13, 18, and 21 was 73.81% (62/84 patients).

During the period of analysis, 22 children (26.19%) were born by cesarean section and 4 cases (4.76%) were premature. Although the sample was small, these were presented heterogeneously but mainly in patients with trisomy 13.

In the case of other viable structural chromosomal changes, constitutive heterochromatins (8.33%) were the most frequent and affected chromosomes 9 (7.14%) and 16 (1.19%). Sex chromosome aneuploidies were only identified in girls (n = 11) (Table 1).

The most frequent clinical characteristics in children with DS (N = 57) were Down facies (45.61%), macroglossia (19.29%), heart murmur (17.54%), and single palmar crease (10.52%). In girls with sexual aneuploidy (n = 11), the most frequent clinical characteristics were short neck (18.18%) and swollen feet/hands (18.18%).

Neonatal age (19 ± 44.9 months, 95% CI, 7.4-30.6, *P* = .001) and paternal age (49 ± 9 years, 95% CI, 37.1-42.5, *P* = .025) showed a significant correlation with the presence of sexual (maternal and paternal age with DS, *P* < .05) and autosomal aneuploidy. Also, a significant correlation was found between height (93.4 ± 17.6 cm, 95% CI, 37.6-149.2) and aneuploidies (*P* = .012), fundamentally autosomal (*P* = .004). Likewise, a significant correlation was found between the presence of aneuploidies and gestational age (30 ± 15.4 weeks, 95% CI, 23.2-36.6, *P* = .001). No correlation was found between the presence of aneuploidy and weight (*P* = .235), gender (*P* = .489), maternal age (37 ± 9 years, 95% CI, 34.1-39.9, *P* = .152), or type of delivery (*P* = .624) (Table 2).

With regard to the patients with sexual aneuploidies (n = 11), 8 (72.72%) reported short stature. Of the 4 cases with Turner’s syndrome (45,X), only 1 patient (25%) presented short stature. On the other hand, short stature affected 5/7 (71.43%) of the children, with alteration of the constitutive chromatin. This structural alteration was presented as a unique finding for these patients.

**Figure 2. A-D.**
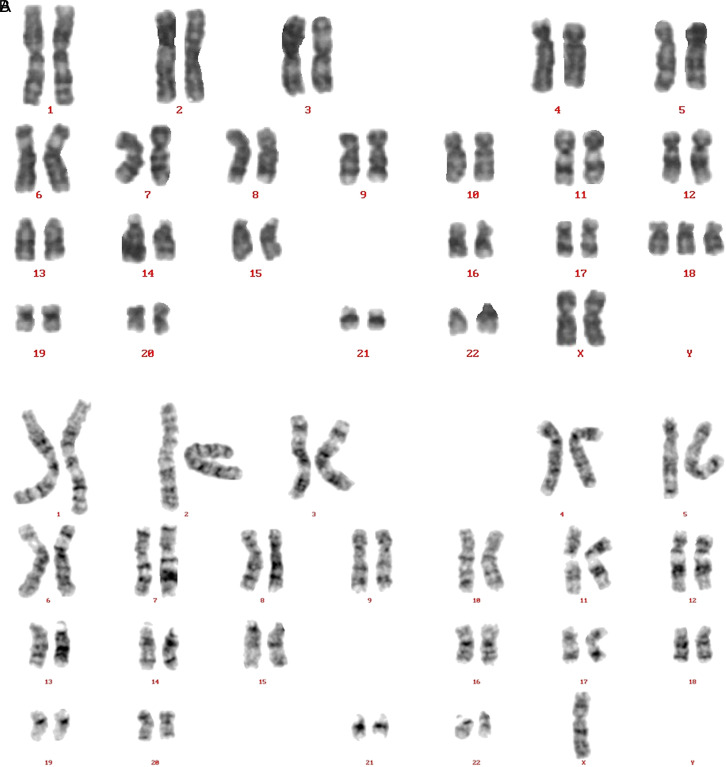

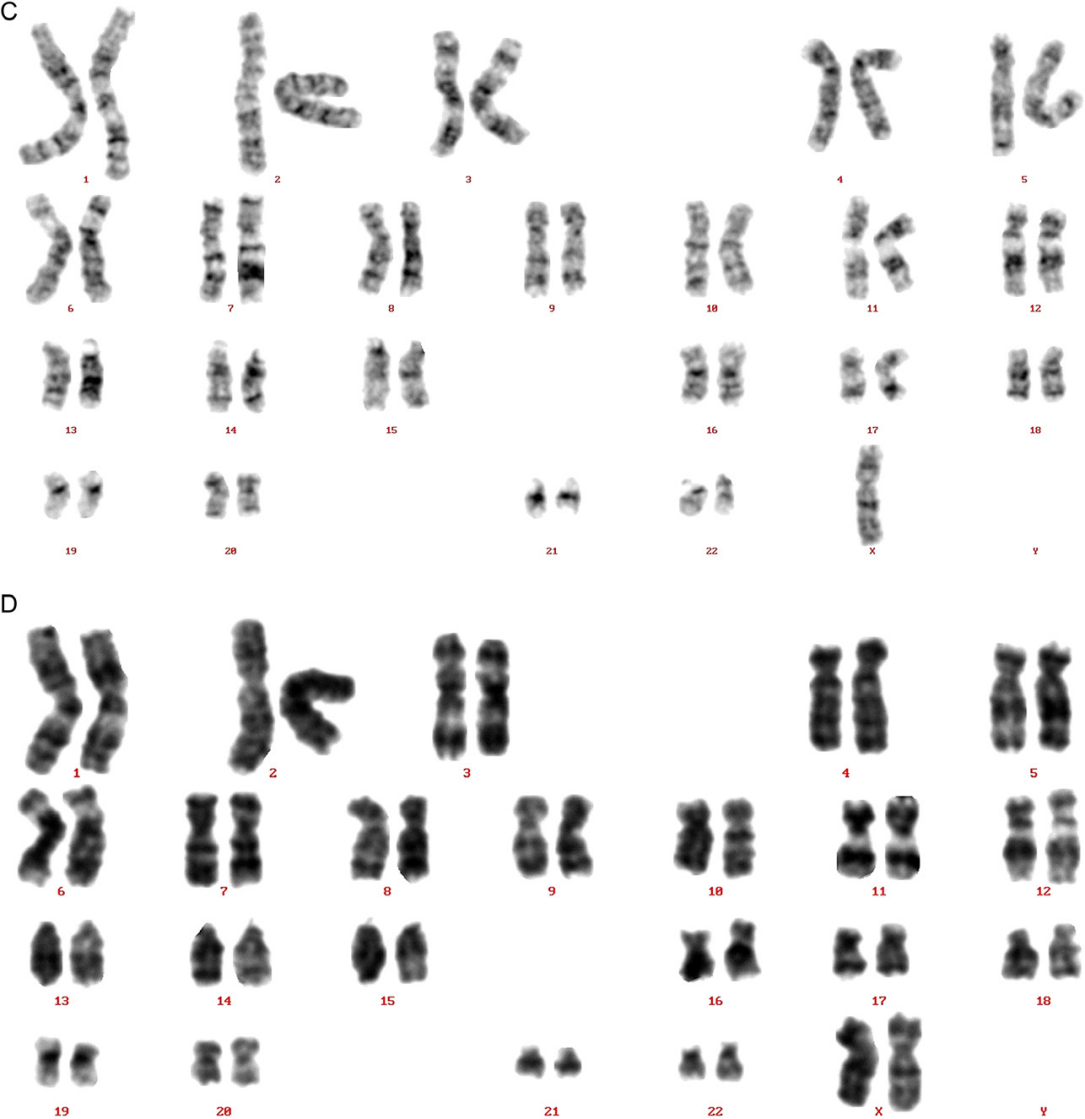
Karyotypes of patients included in the study. A. Normal woman (ID: 0245, 46,XX). B. Woman with free trisomy 21 (ID: 0035, 47,XX,+21). C. Woman with Turner’s syndrome (ID: 0105, 46,X). D. Woman with free trisomy 18 (ID:0024, 47,XX,+18). GTG banding, 100×.

Finally, with respect to the diagnostic contribution of the family tree, the 49 cases in which this information was available (58.33%) were enough to determine whether or not there was a correlation with the presence of aneuploidies. The number of siblings was not significantly associated with the presence of these alterations (*P* = .081). In 16/84 cases (19.04%), the father was 5.3 years older than the mother, and in 5/84 (5.95%) cases, the mother was 7 years older than the father. Only 1 case out of the total number of free trisomies 13 presented a family history with DS (with respect to the 49 cases that had an available family tree information). Figure 2 shows the karyotypes of patients with autosomal and sex chromosome aneuploidies.

## Discussion

In the present study, 84 Peruvian patients with autosomal and sex chromosome aneuploidies were analyzed, and it was found that trisomy 21 was the most frequent (67.85%). In addition, paternal age, gestational age, height, and newborn’s age were significantly correlated with the presence of aneuploidies. On the other hand, short stature did not present significance in the diagnosis of Turner’s syndrome, but, on the contrary, it was significantly associated with alterations in constitutive heterochromatin.

Autosomal aneuploidies were the most frequent in Peruvian children and neonates. A prevalence of 13.09% of sex chromosome aneuploidies was reported, coinciding with the prevalence for these trisomies found in a US nation-wide study in 5 186 504 live births (14.14%, 1.35%, and 3.02% for the trisomies 21, 18, and 13, respectively).^21^

In a European cohort of more than 6 million newborns, the overall prevalence of trisomies 21, 18, and 13 was 22, 5, and 2 per 1 000 000 births, respectively.^22^ Although the prevalence of live neonates with trisomies 21, 18, and 13 decreased to 11.2%, 1.04%, and 0.48%, respectively, the results in both scenarios were consistent with the present study, showing the same frequency of trisomies.

In this study, the most frequent aneuploidy was DS (67.85%; n = 57). Although there are differences in the prevalence of this syndrome around the world, the global tendency places it as the principal trisomy in all continents, with an estimated percentage of 11% to 22%.^22^ A recent study in more than 5 million live births has shown slight differences in the prevalence of trisomies 21, 18, and 13 between populations^21^; thereby, a prevalence of 16.64% for the Hispanic/Latino population, 14.18% for Caucasian Americans, and 11.01% for African Americans was reported. These values show that there is a higher risk of trisomy 21 in the Latino population. This unusual increase may be due to advanced maternal age, educational limitations, and lack of access to prenatal diagnosis and follow-up.^23,24^

Meiotic nondisjunction of chromosome 21 during gametogenesis causes free trisomy 21, which is mainly related to maternal meiosis I, maternal age (29 ± 6 years), and father’s age (35 ± 4 years) to a lesser extent.^25^ In this study, the father's’ (37 ± 8 years ) and mothers’ (35 ± 8 years) average age of children's with free trisomy were represented above the average age for this condition. Both ages were significantly associated with chromosomal abnormalities (*P* < .05).

Since 95% of trisomy DS cases are due to maternal chromosomal nondisjunction, maternal age (≥35 years) could be the main factor associated with this disease.^9,10^ In this study, we determined an association between maternal age and DS (*P* < .05). However, there are other factors, such as reproductive performance, father’s age, maternal periconceptional tobacco use, and long-term use of oral contraceptives, which could play a role in the development and prognosis of DS. Further studies are needed to evaluate the clinical implications of these factors, since we report an association between DS and paternal age in the contribution to risk.^11,12^

Advanced maternal age led to an increase in trisomies 13 and 18 in a study carried out in United Kingdom and Australia on 4.5 million neonates.^26^ Sixty-seven percent and 45.9% of cases of trisomies 18 and 13 have been reported in mothers >35 years of age.^27^ These are the 2 most frequent trisomies after trisomy 21 according to previous studies.^13-15,21,22^ These results concur with our findings that place trisomies 12 and 18 after DS in Peruvian neonates.

Also, the global prevalence of trisomy 18 is 1 per 6000 to 8000 newborns, affecting more neonates of elderly mothers with a high mortality due mainly to congenital heart disease.^13,28^ Same as for DS, there are almost no population differences in the prevalence of trisomy 18 (2.99% in Caucasian population vs. 2.97% in Hispanics/Latinos).^21^ However, in some countries (Argentina, Brazil, and Israel), a higher probability of mortality during the first week of life has been reported.^29,30^ Our study in Peru has determined 4 (4.76%) cases of trisomy 18, and all died after 1 year of follow-up, showing the high mortality of this aneuploidy.

In addition, constitutive heterochromatin, which mainly affects chromosomes 9 (7.14%) and 16 (1.19%), is also prominent. Although chromosome 9 heterochromatin has been associated with reproductive problems^31^ and longevity traits,^32^ these findings are considered benign. Metagenomics and follow-up studies of neonates with these chromatin variants are warranted, as there are many diseases that may be associated with changes in gene expression at the chromatin level (i.e., autism spectrum disorder and nonsyndromic deafness on chromosomes 16 and 9, respectively).

In terms of sex chromosome aneuploidy, X chromosome monosomy was the most common (4.76%). Approximately 1 in 2000 girls is reported to be born with this type of aneuploidy.^33^ In many communities around the world, approximately 45% of Turner’s syndrome cases have a classic karyotype (45,X) followed by mosaic (i.e., 45,X/46,XX).^34^ These results are consistent with the frequencies of X monosomy reported in this study and suggest that both manifestations can be evidenced by common phenotypic traits, such as short stature and short neck.

This study had the following limitations: (i) the 84 patients included represent a small sample; consequently, it is necessary to carry out prospective studies that include a larger number of patients in order to determine how these clinical and demographic characteristics could vary between population groups, since in this study a single-center analysis was performed; (ii) we did not use gene expression tests, since the diagnosis was focused on chromosomal evaluation of patients. Therefore, these results should be completed using gene expression tests mainly in sex chromosome aneuploidies; (iii) this study analyzed the aneuploidies in patients from the Peruvian capital city, Lima; therefore, it is necessary to develop studies on a national scale to determine risk factors for aneuploidies in neonates and children; and (iv) although we evaluated neonatal characteristics, the results should be corrected for parental age and expanded with pre- and postnatal studies.

In conclusion, our results suggest that more than half of the children and neonates presented DS, the most common form of autosomal aneuploidy. Furthermore, these patients exhibited phenotypic features consistent with chromosomal alterations. Among sex chromosome aneuploidies, Turner’s syndrome is the most common, with clinical features consistent with karyotypes, and short stature is present in patients with heterochromatin 9 and 16 .

## Figures and Tables

**Table 1.** Baseline Results of Patients with Sex Chromosome and Autosomal Aneuploidies in Lima, Peru

Aneuploidies^†^	Female	Male	Total
*Sex chromosome aneuploidies*			
46,XX,del(X)	1 (1.19)	….	1 (1.19)
45,X	4 (4.76)	….	4 (4.76)
mos 45,X/46,XX^¶^	2 (2.38)	….	2 (2.38)
mos 45,X/46,X,i(X)(q10)	1 (1.19)	….	1 (1.19)
47,XXX	1 (1.19)	….	1 (1.19)
46,X,i(X)	1 (1.19)	….	1 (1.19)
mos 45,X/46,XY	1 (1.19)	….	1 (1.19)
*Autosomal aneuploidies^‡^*				* Down syndrome*			
Free trisomy	28 (33.33)	24 (28.57)	52 (61.90)
Robertsonian*	1 (1.19)	3 (3.57)	4 (4.76)
Mosaicism^§^	1 (1.19)	….	1 (1.19)
Edwards syndrome***	2 (2.38)	2 (2.38)	4 (4.76)
Patau syndrome***	….	1 (1.19)	1 (1.19)
* Others*			
46,XX,r(15)(q26.2)	1 (1.19)	….	1 (1.19)
47,XY,mar+	….	1 (1.19)	1 (1.19)
Heterochromatin**	4(4.76)	3 (3.57)	7 (8.33)
46,XY,t(13;14)	….	1 (1.19)	1 (1.19)
46,XX,14psth+,15pst	1 (1.19)	….	1 (1.19)
Total	49 (58.33)	35 (41.66)	84 (100)

Data are presented as n (%).

*Robertsonian translocation includes a case of 46,XX,t(14;21)(q10:q10), and 3 cases correspond to 46,XY,t(21;21)(q10:q10).

**Four (4.76%) cases of 9qh−, 2 (2.38%) cases of 9hq+, and a single case (1.19%) of 16qh+ were identified.

***Reported only for free trisomy.

^¶^The average of metaphases counted was mos 45,X [49]/46,XX [20]. ^‡^The most frequent trisomies represented 73.80% (62 cases).

^§^ mos 46,XX[17]/47,XX,+21 [15].

^†^ *P* < .001 (significant).

**Table 2.** Associations Between Aneuploidies and Main Characteristics of Newborns and Parents

Characteristics		Aneuploidy		Both
		Autosomal	Sex Chromosome	
Newborn	Newborn’s age	*.001*	*<.001*	***.001***
	Height	*.004*	*.011*	***.012***
	Weight	*.551*	*.084*	*.235*
	Gender	*.306*	*.745*	*.489*
Parents	Paternal age	*.013*	*.023*	***.025***
	Gestational age	*<.001*	*<.001*	***.001***
	Maternal age	*.077*	*.100*	*.152*
	Type of delivery	*.228*	*.341*	*.624*
	Number of siblings	*.078*	*.061*	*.081*
	Previous abortions	*.196*	*.097*	*.101*

*P*-values are indicated, *P *< .05 in bold.
